# Different Behavior of Enteric Bacteria and Viruses in Clay and Sandy Soils after Biofertilization with Swine Digestate

**DOI:** 10.3389/fmicb.2017.00074

**Published:** 2017-01-31

**Authors:** Gislaine Fongaro, María C. García-González, Marta Hernández, Airton Kunz, Célia R. M. Barardi, David Rodríguez-Lázaro

**Affiliations:** ^1^Laboratório de Virologia Aplicada, Departamento de Microbiologia, Imunologia e Parasitologia, Universidade Federal de Santa CatarinaFlorianópolis, Brazil; ^2^Instituto Tecnológico Agrario de Castilla y LeónValladolid, Spain; ^3^Departamento de Ingeniería Agrícola y Forestal, Tecnología de los Alimentos, Escuela Técnica Superior de Ingenierías Agrarias, Universidad de ValladolidPalencia, Spain; ^4^Embrapa Suínos e AvesConcórdia, Brazil; ^5^Microbiology Section, Department of Biotechnology and Food Science, Faculty of Sciences, Universidad de BurgosBurgos, Spain

**Keywords:** swine digestate, clay and sandy soils, biofertilization, biomarkers, management

## Abstract

Enteric pathogens from biofertilizer can accumulate in the soil, subsequently contaminating water and crops. We evaluated the survival, percolation and leaching of model enteric pathogens in clay and sandy soils after biofertilization with swine digestate: PhiX-174, mengovirus (vMC_0_), *Salmonella enterica* Typhimurium and *Escherichia coli* O157:H7 were used as biomarkers. The survival of vMC_0_ and PhiX-174 in clay soil was significantly lower than in sandy soil (*i*T_90_ values of 10.520 ± 0.600 vs. 21.270 ± 1.100 and 12.040 ± 0.010 vs. 43.470 ± 1.300, respectively) and PhiX-174 showed faster percolation and leaching in sandy soil than clay soil (*i*T_90_ values of 0.46 and 2.43, respectively). *S*. *enterica* Typhimurium was percolated and inactivated more slowly than *E. coli* O157:H7 (*i*T_90_ values of 9.340 ± 0.200 vs. 6.620 ± 0.500 and 11.900 ± 0.900 vs. 10.750 ± 0.900 in clay and sandy soils, respectively), such that *E. coli* O157:H7 was transferred more quickly to the deeper layers of both soils evaluated (percolation). Our findings suggest that *E. coli* O157:H7 may serve as a useful microbial biomarker of depth contamination and leaching in clay and sandy soil and that bacteriophage could be used as an indicator of enteric pathogen persistence. Our study contributes to development of predictive models for enteric pathogen behavior in soils, and for potential water and food contamination associated with biofertilization, useful for risk management and mitigation in swine digestate recycling.

## Introduction

Global demand for fertilizer nutrients, including nitrogen, phosphorus, and potassium, is expected to reach 200,500,000 tons by 2018, and food demand for human consumption and livestock feed is expected to increase by 60–110% by 2050. Food production sustainability is therefore essential, and will require nutrient and water recycling under appropriate conditions of health and safety (Tilman et al., 2001; Food and Agriculture Organization of the United Nations [FAO], 2014). Swine manure is biomass suitable for biogas production in anaerobic biodigesters (AB) and the final digestate is used as agricultural biofertilizer. Digestate recycling is ecologically and economically viable, because it can simultaneously biofertilize and irrigate various crops, such as maize, wheat, and soybean, satisfying some or even all nitrogen and phosphorus requirements (Hansen et al., 1998; Topp et al., 2009).

Human and animal diseases associated with soil can be caused by exposure to or ingestion of indigenous pathogens of the normal soil microbiota, food contaminated with enterotoxins and neurotoxins, or enteric pathogens from human or animal excreta (Santamaría and Toranzos, 2003). Swine digestate can contain high levels of enteric pathogens that may contaminate soil, water, and foods (Khetsuriani et al., 2006; Topp et al., 2009). Its use in agriculture is therefore an issue for global “One Health” as it may affect humans and animals and, indeed, the environment more generally. The enteric pathogens present in biofertilizers can accumulate in soil (Gerba and Smith, 2005). Microbial models can be used to assess the behavior of enteric pathogens, including their survival, flow, propagation, leaching, percolation, and inactivation (Langlet et al., 2008; Boudaud et al., 2012). Bacteriophages, such as somatic coliphages (e.g., PhiX-174), f-specific RNA phages (e.g., MS2), and mengovirus (vMC_0_) have been used as models for human and animal enteric viruses (Langlet et al., 2008; Boudaud et al., 2012). Similarly, enteric bacteria, particularly coliform and *Salmonella* ssp., are widely used as biomarkers of fecal contamination. *Salmonella* is one of the most prevalent bacterial pathogens; it is zoonotic and has high survival rates in environment (Griffith et al., 2006; Venglovsky et al., 2006; European Food Safety Authority, and European Centre for Disease Prevention, and Control, 2015).

There have been few rigorous analyses of how enteric microorganisms behave in soil after being introduced by biofertilization, or of rates of environmental survival and propagation, leaching and percolation in different types of soil. Such analyses are required before swine-derived biofertilizers can be widely exploited agriculturally. The movement of enteric bacteria through soils, and the percolation of microorganisms in soils more generally, depend largely on the water saturation state (Artz et al., 2005). European Regulation (EC) 1069/2009, addressing health aspects of animal by-products and derived products not intended for human consumption, requires that *Salmonella* spp. to be below the detection threshold (25–50 g samples of biofertilizer scoring negative) and fewer than 1000 *E. coli* CFU g^-1^ of wet weight of biofertilizer (EC, 2009). In some countries, for example Brazil, there are no specific domestic or international rules for recycling animal by-products (Kunz et al., 2009). There is an evident need for the rigorous assessment of the fate of important enteric microorganisms after biofertilization of different types of soils. The development of appropriate bio-models for diverse environmental scenarios (survival, percolation, and leaching) would be valuable. However, there are currently no reports in the literature suggesting models that could be used to investigate the stability, percolation, and leaching of enteric pathogens, simultaneously. There has also been no comparison of the behaviors of enteric viruses and bacteria in sandy and clay soils in *ex situ* studies.

We aimed to (i) evaluate the survival and percolation of several major enteric microorganisms and to (ii) select the most suitable enteric microorganisms to serve as biomarkers of percolation and leaching after biofertilization with swine digestate. We spiked swine digestate with several model microorganisms (PhiX-174, vMC_0_, *Salmonella enterica* Typhimurium and *E. coli* O157:H7) and applied samples to clay and sandy soils. We then evaluated survival, percolation and leaching of the added microorganisms.

## Materials and Methods

### Swine Digestate and Soil

Digestate from mesophilic AB (35 ± 2°C) was collected from a pig farm in Salamanca, Spain. Clay and sandy soil were collected in agriculture fields, in Valladolid, Spain, that were not cultivated. Physicochemical characteristics of the swine eﬄuent and soils were determined: chemical oxygen demand (COD), total solid (TS), nitrogen (TN) and phosphorus (TP) and pH for swine eﬄuent; and texture, TN, organic carbon (OC) and matter (OM), carbonate, total carbon (TC), extractable sodium (ES), potassium (EP), calcium (EC), and magnesium (EM), percentage of clay and sand, pH and conductivity for soils according to APHA (2002) (**Table 1**). The OM, TC, EP, EC, EM, pH, and conductivity were all significantly higher in clay soil than in sandy soil.

**Table 1:** Physicochemical characteristics of clay and sandy soil used in this study before and after biofertilization, and of the digestate used for biofertilization^a^.

	Before biofertilization		After biofertilization	
	Clay soil	Sandy soil	Clay soil	Sandy soil
TN (%)	0.07 ± 0.01	0.02 ± 0.01	0.15 ± 0.11	0.08 ± 0.04
OC (%)	0.67 ± 0.08	0.23 ± 0.04	1.45 ± 0.03	0.30 ± 0.03
OM (%)	1.16 ± 0.09^∗^	0.42 ± 0.05	2.57 ± 0.06	0.71 ± 0.02
Carbonate (%)	<3.00	<3.00	<3.00	<3.00
TC (%)	0.82 ± 0.05^∗^	0.27 ± 0.03	1.23 ± 0.15	0.32 ± 0.01
ES (mg g^-1^)	<0.02	<0.02	0.08 ± 0.01	0.05 ± 0.01
EP (mg g^-1^)	0.34 ± 0.05^∗^	0.033 ± 0.01	0.79 ± 0.01	0.15 ± 0.01
EC (mg g^-1^)	2.37 ± 0.15^∗^	<1.60	6.39 ± 1.20^∗^	<1.60
EM (mg g^-1^)	0.23 ± 0.01^∗^	<0.08	0.73 ± 0.09	<0.08
Clay (%)	32.2 ± 0.20^∗^	2.1 ± 0.41	34.8 ± 0.30	3.41 ± 0.10
Sand (%)	12.0 ± 1.25^∗^	95.50 ± 1.41	13.0 ± 1.40	94.0 ± 1.47
pH	8.33 ± 0.85^∗^	6.71 ± 0.55	8.62 ± 0.40	6.71 ± 0.02
Conductivity (mS/cm)	97.00 ± 3.20^∗^	12.21 ± 1.10	282.32 ± 4.10	114.3 ± 0.12

**Swine eﬄuent from mesophilic anaerobic biodigesters (AB)**				

Total COD (mg L^-1^)	42,246 ± 375			
TS (mg L^-1^)	32,130 ± 267			
TN (mg L^-1^)	5,936 ± 380			
TP (mg L^-1^)	523 ± 43			
pH	7.30 ± 0.20			

*^a^Mean ± Standard deviation.*

*^∗^Significant difference (*p* < 0.05).*

### Model Microorganisms

*Escherichia coli* O157:H7 strain CECT 4267, *S. enterica* subsp. *enterica* serovar Typhimurium strain ATCC 14028, the avirulent genetically modified mengovirus strain M (vMC_0_) ATCC VR-1597, and PhiX-174 were used as model enteric pathogens. Bacterial stocks were prepared in nutrient broth, for 14 h at 37°C, as described by Magri et al. (2013). PhiX-174 was propagated in the host *E. coli* strain ATCC 13706 as described by International Organization For Standardization [ISO] 10705-2:2000 (2000) and vMC_0_ stocks were prepared from an infected HeLa cell line (ATCC CCL-2TM). Cells were cultivated in six-well tissue culture plates at a density of 3.0 × 10^6^ cells/mL and were incubated at 37°C in 5% CO_2_ for 24 h with vMC_0_ stocks, as described by Costafreda et al. (2006).

*Salmonella enterica* Typhimurium was counted using the method of Magri et al. (2013) and *E. coli* by the ISO 4832:2006 method. Colonies were counted and the results are expressed in colony forming units (CFU). PhiX-174 was titred in agar on *E. coli* ATCC 13706, by the double agar layer method (International Organization For Standardization [ISO] 10705-2:2000, 2000). Plaques were counted and the results are expressed in plaque forming units (PFUs). Infectious vMC_0_ was titred by plaque assay (PA), as described by Ernest et al. (1970). For PA, briefly, 1 mL or g of sample was treated with 10 U/mL penicillin, 10 μg/mL streptomycin, and 0.025 μg/mL amphotericin B and diluted with 9 mL of saline buffer and 0.25 mL samples used to inoculate HeLa cells. HeLa cells were cultivated in six-well tissue culture plates at a density of 3.0 × 10^6^ cells/well and were incubated at 37°C in 5% CO_2_ for 24 h. Inoculated cells were plated and incubated at 37°C for 5–7 days. The plaques were counted macroscopically and the results are expressed in PFUs.

### Preparation and Analysis of Microcosms of Biofertilized Soils

#### Microbial Survival Assay

We studied the survival of the model enteric microorganisms over 120 days in biofertilized clay and sandy soils. All experiments were performed in triplicate using sentinel chambers, as described by Schwarz et al. (2014). Sentinel chambers were constructed using 3.5 mL tubes with pore sizes of 0.2 μm (Life Sciences, New York, NY, USA) and membrane lids (Eppendorf Lid-Bac membrane lids, Eppendorf, Germany) to close the top of the columns. The pore size and electric charge was sufficiently large to allow exchange of gas and moisture without the loss of bacteria or viruses.

Biofertilized soils containing 7 log_10_ CFU g^-1^ and 5 log_10_ PFU g^-1^ of the model enteric bacteria and viruses, respectively, were used to fill sentinel chambers and placed vertically in clay and sandy soil microcosms (10–20 cm of depth) at 23 ± 2°C (environmental average temperature during the spring period, when soils are fertilized in agricultural practice). The amount of swine eﬄuent used in the experiment mimicked that usually applied for corn and wheat crops (50 m^3^/hectare).

Soils samples were collected after: 1, 4, 10, 20, 30, 40, 50, 60, 80, and 120 days. On each sampling date, two sentinel chambers were randomly selected for each soil microcosm, and evaluated. Inactivation was considered statistically significant if the count (or titre) declined by >2 log_10_ with *p* < 0.05 (ANOVA, GraphPad Prism 5.0, EUA). Also, *i*T_90_ values in soils were calculated until 120 days after biofertilization.

#### Microbial Percolation Assay

Polyvinyl chloride tubes (PVC tubes), 60 cm long and 30 cm in diameter, were closed with a cap at the bottom, and arranged horizontally in an air-conditioned environment (22 ± 2°C). The soils were placed in the tubes, in the same order they were removed from the farm (until 60 cm deep). All tubes were then placed vertically and were left undisturbed for a week to allow the soil to settle. Then, the top 4 cm of soil was discarded to provide space for the swine eﬄuent (biofertilization). The clay soil density was 1.6 and the sandy soil 1.3 g cm^-3^. The soils in the PVC tubes were biofertilized by spraying with swine eﬄuent from mesophilic AB containing 7 log_10_ CFU g^-1^ of the model enteric bacteria and 5 log_10_ PFU g^-1^ of the model viruses. All microorganisms were applied together, mimicking contamination in biofertilizers in practice. The amount of swine eﬄuent used was equivalent to that usually applied for corn and wheat cultivation (50 m^3^/hectare).

Percolation of the enteric microorganisms was followed by collection of 1 g samples of soil at depths of 10, 20, 30, 40, and 50 cm by making holes of 1 cm in diameter in the PVC tubes using a sterile probe. Samples were collected 0, 0.12, 0.24, 0.5, 1, 2, 4, 8, 15, and 20 days after biofertilization.

The initial microbial concentration in layers 10 cm deep and the final concentration 50 cm deep were used to calculate percolation. The soil layers (10, 20, 30, 40, and 50 cm depths) were grouped according to the distribution of microorganisms over time, and the decimal reductions value (*r*T_90_ days) and decimal increase value (*i*T90 days) caused by the percolation action were calculated for each clay and sandy soil.

#### Microbial Leaching from Biofertilized Soils after Rain

For leaching assays, 120 days after biofertilization, the soil microcosms were subjected to 300 mm of rain (collected natural rain, 150 mm h^-1^, applied over 2h). A sterile collector tube, with capacity of 100 mL, was placed under the soil microcosms, horizontally, allowing the collection of leaching liquid (drained through a lower opening). Liquid drained from soils was collected 2, 4, 8, 12, 24, 36, and 48 h after the rain and the enteric microorganisms counted.

### Statistical Analysis

Nonparametric analysis of variance (ANOVA) (Kruskal–Wallis) in Statistic 7.0 was used to evaluate the differences in the physicochemical parameters of the soils. The times required for a 10-fold reduction in enteric microorganism counts by inactivation and enteric microorganism leaching from biofertilized soils were calculated considering linear or logarithmic regression curves. Statistical one-way ANOVA was used to evaluate differences between the groups, using a 95% confidence level, followed by the Bonferroni’s Multiple Comparison Test, and Pearson’s correlation analysis was used as necessary (GraphPad Prism 5.0). The critical *p*-value for the test was set at ≤0.05.

## Results

### Survival of the Model Microorganisms

**FIGURE 1:**
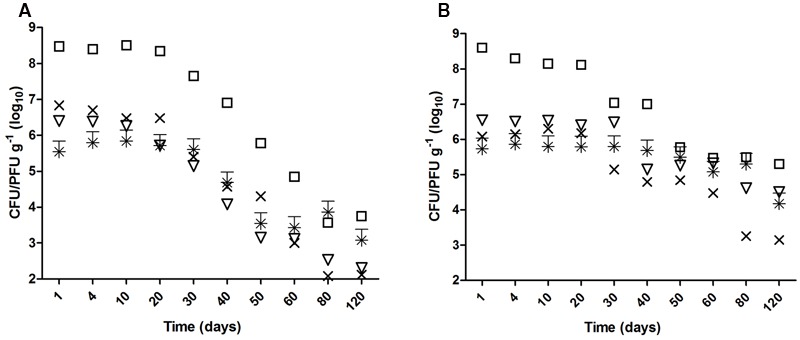
**Survival of enteric microorganisms in biofertilized (A)** clay and **(B)** sandy soil 120 days after biofertilization with contaminated swine digestate. (□) *Escherichia coli* O157:H7, (×) *S*. Typhimurium, (^∗^) PhiX-174, and (▽) vMC_0_.

*Escherichia coli* O157:H7, *S*. *enterica* Typhimurium were significantly reduced (>2 log_10_; *p* < 0.05) after 40 days in biofertilized soil of both types (**Figure 1**). However, whereas the time for a significant reduction in clay soils was similar for the two viruses selected in this study (40 days for vMC_0_ and 50 days for PhiX-174), the times were longer in sandy soils (60 days for vMC_0_, and no significant reduction of PhiX-174 after 120 days) (**Figure 1**). Similarly, the *i*T_90_ values were higher in clay than in sandy soils for all the microorganisms (6.62 vs. 10.75 for *E. coli* O157:H7, 9.34 vs. 11.90 for *S*. *enterica* Typhimurium, 10.52 vs. 21.27 for vMC_0_, and 12.04 vs. 43.47 for PhiX-174) (**Table 2**).

**Table 2:** Mean *i*T_90_ (decimal inactivation), inactivation coefficients (*k*), and multiple-correlation coefficients (*r*^2^) for soils after biofertilization^a^.

	-*k*	*i*T_90_ days	*r*^2^	-*k*	*i*T_90_ days	*r*^2^
	Clay soil			Sandy soil		
*Escherichia coli* O157:H7	0.151 ± 0.020^∗1^	6.620 ± 0.500	0.870 ± 0.040	0.093 ± 0.030	10.750 ± 0.900	0.880 ± 0.040
*S*. Typhimurium	0.0107 ± 0.030^∗1^	9.340 ± 0.200	0.990 ± 0.050	0.084 ± 0.020	11.900 ± 0.900	0.810 ± 0.050
vMC_0_	0.095 ± 0.030^∗2^	10.520 ± 0.600	0.880 ± 0.020	0.047 ± 0.010	21.270 ± 1.100	0.930 ± 0.020
PhiX	0.083 ± 0.010^∗2^	12.040 ± 1.300	0.820 ± 0.030	0.023 ± 0.010	43.470 ± 1.300	0.840 ± 0.030

*^a^Mean ± Standard deviation.*

*^∗^Significant difference (*p* < 0.5). Natural number indicates the cluster.*

### Percolation of the Selected Model Microorganisms

**FIGURE 2:**
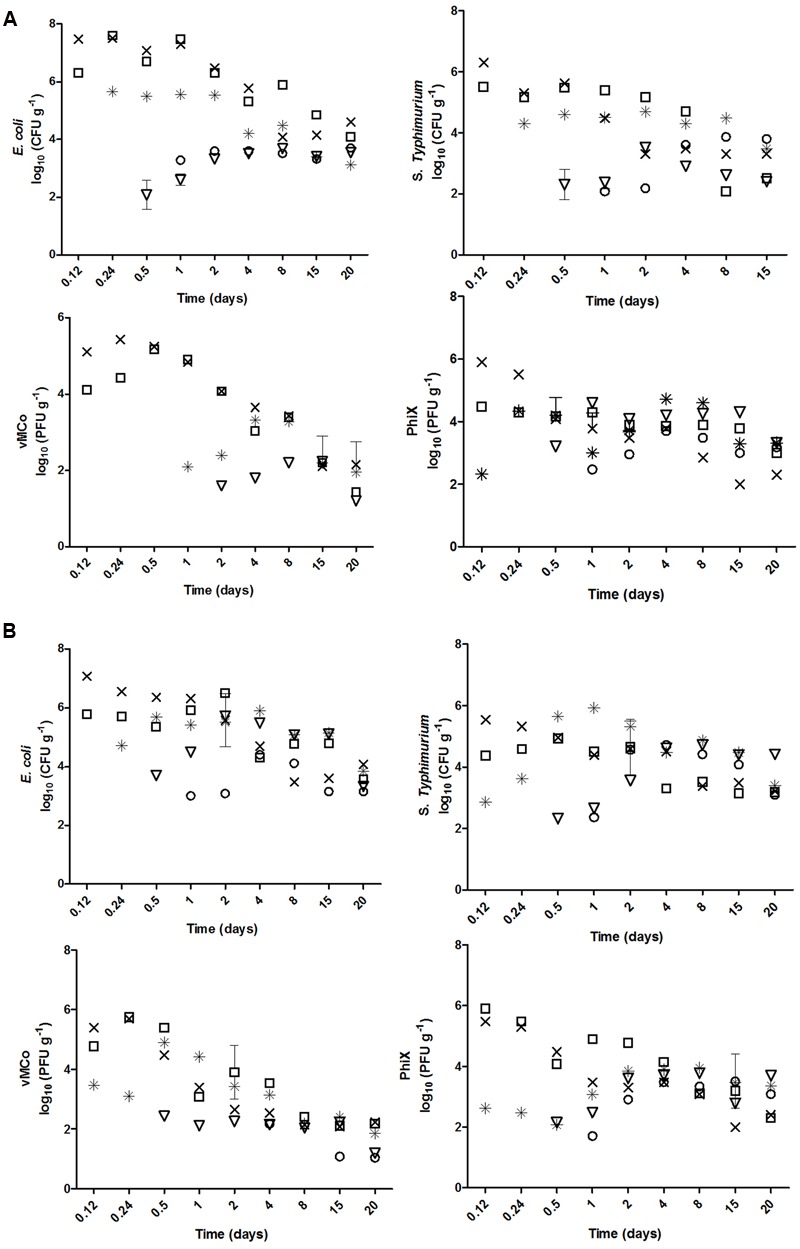
**Percolation of *E. coli* O157:H7, *S*. Typhimurium, vMC_0_ and PhiX-174 (×) 10, (□) 20, (^∗^) 30, (▽) 40, and (○) 50 cm deep in biofertilized (A)** clay and **(B)** sandy soil.

Significant percolation was observed for all four microorganisms and both soil types (**Figure 2**). The mean percolation was similar in the two types of soil for each microorganism: log_10_ 3.80 vs. log_10_ 3.92 for *E. coli* O157:H7, log_10_ 2.60 vs. log_10_ 2.45 for *S. enterica* Typhimurium, log_10_ 4.2 vs. log_10_ 4.2 for vMC_0_, and log_10_ 2.8 vs. log_10_ 2.32 for PhiX-174 in clay and sandy soils, respectively. *E. coli* O157:H7 and vMC_0_ percolated significantly more than the other two microorganisms in both soils (*p* < 0.05) (**Figure 2**).

The percolation of the microorganisms from the more superficial (10, 20, and 30 cm) to the deeper (40 and 50 cm) layers was linear, whereas the increase of the concentration of microorganisms in the deeper layers (40 and 50 cm) was logarithmic. The percolation from the upper layers to the deeper ones was not significantly different for the four microorganisms (*p* > 0.05) in sandy soil, the percolation of PhiX-174 and *E. coli* O157:H7 were significantly slower and faster (*p* < 0.05), respectively, in clay soil (**Table 3**).

**Table 3:** Mean *r*T_90_ (decimal reductions) and *i*T_90_ (decimal increase) by percolation per day, respective coefficients (*k*) and multiple-correlation coefficients (*r*^2^) for clay and sandy soil after biofertilization.

	Group (cm)	*K* (days)	*r*^2^	*r*T_90_ (days)	*i*T_90_ (days)	Model
***A. Clay soil***
*E. coli* O157:H7	10–20	0.61	0.94	1.62	–	Linear
	30–40–50	1.11^∗1^	0.76	–	0.90	Logarithmic
	30–40–50	2.35^∗2^	0.82	–	0.42	Logarithmic
*S*. Typhimurium	10–20	0.57	0.92	1.73	–	Linear
	30–40–50	0.60	0.76	–	1.64	Logarithmic
vMC_0_	10–20	0.41	0.95	2.84		Linear
	30–40–50	0.42	0.91	–	0.53	Logarithmic
PhiX	10–20	0.27	0.95	3.58	–	Linear
	30–40–50	0.41	0.72	–	2.43	Logarithmic
***B. Sandy soil***
*E. coli* O157:H7	10–20	0.47	0.91	2.12	–	Linear
*S*. Typhimurium	10–20	0.42	0.91	2.38	–	Linear
	30–40–50	1.10	0.84	–	0.90	Logarithmic
vMC_0_	10–20	0.35	0.79	2.85	–	Linear
	30–40–50	0.49	0.67	–	2.04	logarithmic
PhiX	10–20–40	0.41	0.95	2.43	–	Linear
	40–50	2.22^†^	0.91	–	0.45	Logarithmic

*^∗^Significant difference (*p* < 0.05). Natural number indicates the cluster.*

*^†^Significant difference (*p* < 0.05).*

#### Leaching of the Selected Model Microorganisms

**FIGURE 3:**
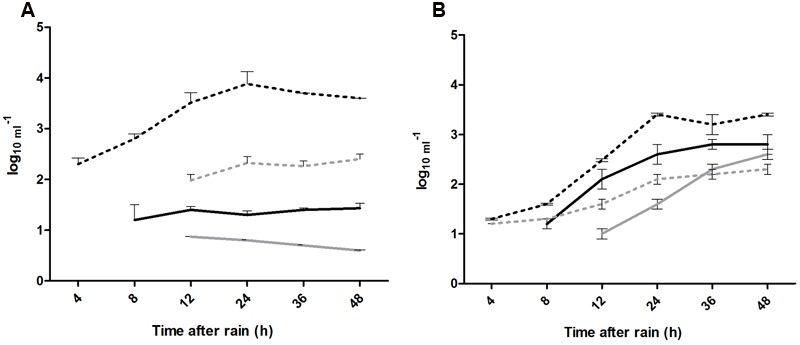
**(A,B)** Leaching of enteric microorganisms over 48 h following application of 300 mm of natural rain. ^∗^Significant difference (*p* < 0.05), where: (---) *S*. Typhimurium, (**---**) *E. coli* O157:H7, (-) vMC_0_, and (**-**) PhiX-174.

The leaching of the enteric bacteria (*E. coli* O157:H7 and *S*. *enterica* Typhimurium) over 48 h was higher in clay soils than in sandy soils (log_10_ 3.81 vs. log_10_ 2.80 and log_10_ 2.24 vs. log_10_ 1.90 per mL, respectively), whereas the leaching of the model viruses (vMC_0_ and PhiX-174) was much higher in sandy soil (log_10_ 1.8 vs. log_10_ 0.7 and log_10_ 2.3 vs. log_10_ 1.34 per mL, respectively) (**Figure 3**).

**FIGURE 4:**
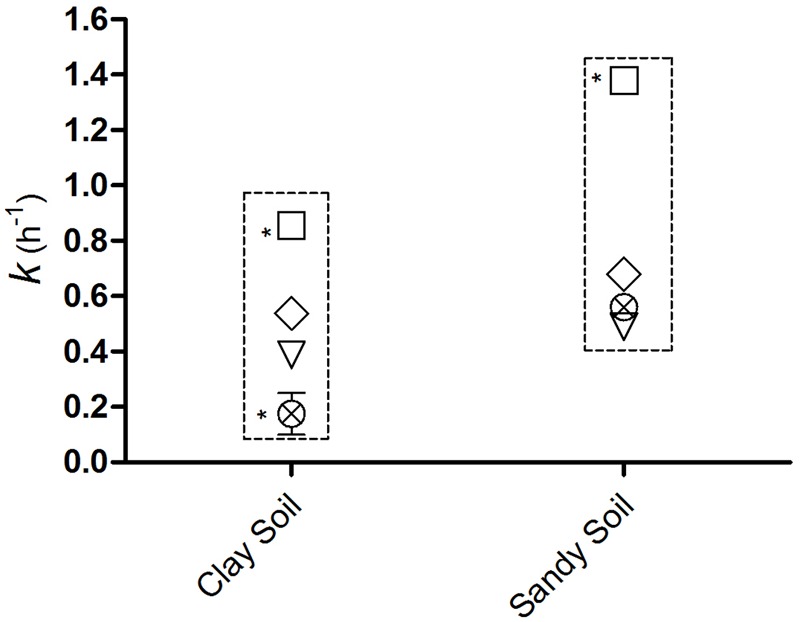
**Coefficients of leaching (*k* h^-1^) of (□) *E. coli* O157:H7, (♢) *S*. Typhimurium, (⊗) PhiX-174, and (∇) vMC_0_ over 48 h following application of 300 mm of natural rain.** ^∗^Significant difference (*p* < 0.05).

The coefficients of leaching (*k* h^-1^) were 0.85 for *E. coli* O157:H7, 0.53 for *S*. *enterica* Typhimurium, 0.38 vMC_0_ and 0.17 for PhiX-174 in clay soils, and 1.3, 0.68, 0.49, and 0.56, respectively, in sandy soils (**Figure 4**). The coefficients of leaching were higher in sandy soils for all four microorganisms, and higher for the two bacteria than the two viruses; the leaching of *E. coli* O157:H7 was significantly highest (*p* < 0.05) in both soils (**Figure 4**).

## Discussion

Soil features, including temperature, pH, clay, organic matter, and salinity content, affect the survival, stability, percolation, and leaching of enteric pathogens present (Kimura et al., 2008). We investigated the survival and percolation of four relevant enteric microorganisms (two bacterial pathogens: *E. coli* O157:H7, and *S. enterica* Typhimurium and two viruses: PhiX-174, vMC_0_) in clay and sandy soils in a model of biofertilization with swine eﬄuent from AB. *E. coli* O157:H7 and *Salmonella* ssp. are major zoonotic enteric bacterial pathogens. *Salmonella* ssp. are often isolated from farmed pigs and poultry, which are the main reservoirs for human infections. These species show high survival rates in the environment (Griffith et al., 2006; Chen and Jiang, 2014). Somatic coliphages and vMC_0_ have been used as models for human and animal enteric viruses. PhiX-174 is stable across a range of temperatures, pHs and UV treatments, and vMC_0_ has been used as model of norovirus, and hepatitis A and E viruses (Costafreda et al., 2006; Rodríguez-Lázaro et al., 2012; Boudaud et al., 2012).

Soils are complex matrixes and some of the compounds present have antimicrobial (virucidal and/or bactericidal) properties when concentrated: examples include malic, lactic and butyric acids, proteases, nucleases, bacterial and fungal metabolites; there may also be microorganism predation (Wiesmann et al., 2007; Dika et al., 2011). The survival, percolation, and leaching of the four microorganisms we studied differed between the two soil types. However, microbial survival and leaching was significantly lower in clay soil than in sandy soil for all the four microorganisms. The organic matter and nutrient contents are higher in clay than sandy soil, and the pH more alkaline. The pH and salt concentration (salt increases conductivity) may directly affect the adsorption capacity of viruses. Increasing the concentration of cations, especially multivalent cations, increases the adsorption capacity, mainly for enteric viruses (Gerba, 1984; Gerba and Smith, 2005; Pepper et al., 2006). The leaching of nutrients is high on sandy soils with poor water retention capacity (Department for Environment, Food and Rural Affairs, 2010). PhiX-174 showed faster percolation and leaching from biofertilized sandy soil than clay soil. The isoeletric point of PhiX-174 is 6.5 (Schijven and Hassanizadeh, 2000) and the pH of clay and sandy soils studied was 8.6 and 6.7, respectively. Therefore, PhiX-174 may have aggregated in the clay soil, hindering its percolation and leaching, whereas this was unlikely to have been the cases in sandy soil.

We report that the survival of the model viruses was significantly longer and correspondingly that microbial inactivation was faster for bacteria (*i*T_90_ days). Bacteriophage PhiX-174 showed the least inactivation the highest *i*T_90_ value of the four microorganisms studied (**Table 2** and **Figure 1**). PhiX-174 may therefore be useful as a biomarker for the study of persistence of enteric pathogens in soils. Enteric viruses can survive for long periods [DNA viruses like human adenovirus decay by only 90% in 180 days in biosolid-amended and un-amended soils (Schwarz et al., 2014)] particularly in soils with a higher moisture content (60–90 days in soils with 10% moisture content and 15–25 days in air-dried soil (Yeager and O’Brien, 1979).

All four microorganisms percolated significantly in both soil types. Thus, a proportion of the microorganisms applied at the surface can be transferred into deeper layers, and the reduction of microbial survival may be, in part, associated to this process. The percolation of the microorganisms was linear, but microbial accumulation in the deeper layers was logarithmic. *S*. *enterica* Typhimurium percolated and was inactivated more slowly than *E. coli* O157:H7, suggesting that it may readily remain in contact with vegetables of short roots, like lettuce. *E. coli* O157:H7 was both transferred to the deeper layers of both soils (i.e., percolation) and leached faster, raising the issue of contamination of surface and groundwater used for human supply and for plant irrigation. *E. coli* O157:H7 and *Salmonella* serovar Typhimurium are able to move through soil with water after rainfall or irrigation and can even reach the groundwater (Artz et al., 2005; Lang and Smith, 2007; Semenov et al., 2009). Field experiments showed that 20% of *E. coli* applied to fields in contaminated slurry was found in drain water (Vinten et al., 2002). Also, our study and previous results reveal the risk of internal contamination of plants by *E. coli* O157:H7 and other coliforms. In view of these observations, international regulations are surprisingly divergent about microbiological criteria for water for human consumption and irrigation (Chart, 2000; Semenov et al., 2009).

We show that *E. coli* O157:H7 has potential as a microbial biomarker for depth contamination and leaching in clay and sandy soils. Note that it is possible that genogroups of *E. coli* different from *E. coli* O157:H7 behave differently in these or other soils.

## Conclusion

We report that survival, percolation, and leaching of enteric microorganisms in biofertilized soils depend on the characteristics of the microbes and the soil. Our findings corroborate previous work suggesting that pH, OM content, soil texture, and rainfall are the principal factors that affect the survival and leaching of microbial pathogens (Demoling et al., 2007). We believe that potential microbial contamination of surface and groundwater by leaching and percolation should be evaluated prior to swine digestate application in agriculture. Finally, our work shows that the bacteriophage PhiX-174 and the bacterial pathogen *E. coli* O157:H7 can be used as biomarkers for studies on microbial persistence and percolation and leaching, respectively. Our findings contribute to the development of predictive models of the behavior of enteric pathogens in different soils and of the potential transfer of pathogens to water and food by biofertilization. Such models would be useful for risk management and mitigation in swine digestate recycling.

## Author Contributions

GF performed most of the experiments and help in the preparation of the manuscript. MG-G discussed the experiments, review the results and help in the preparation of the manuscript. MH discussed the experiments, supervise the experimental work and review the results and help in the preparation of the manuscript. AK review the experimental plan and the results. CB discussed the experiments, review the results and help in the preparation of the manuscript. DR-L designed the experiments, review the results, and prepare the manuscript.

## Conflict of Interest Statement

The authors declare that the research was conducted in the absence of any commercial or financial relationships that could be construed as a potential conflict of interest.
